# Characteristics of in-hospital stroke patients in Sweden: A nationwide register-based study

**DOI:** 10.1177/23969873231182761

**Published:** 2023-06-17

**Authors:** Ilan Ben-Shabat, David Darehed, Marie Eriksson, Jonatan Salzer

**Affiliations:** 1 Department of Clinical Science, Neurosciences, Umeå University, Umeå, Sweden; 2 Department of Public Health and Clinical Medicine, Sunderby Research Unit, Umeå University, Umeå, Sweden; 3 Department of Statistics, USBE, Umeå University, Umeå, Sweden

**Keywords:** In-hospital stroke, intrahospital stroke, in-house stroke, brain, stroke and cerebrovascular disorders, ischemic stroke, hemorrhagic stroke, invasive procedures, surgery and anesthesia, post-operative, reason for hospitalization, clinical epidemiology

## Abstract

**Introduction:**

Few studies have reported the characteristics of patients with in-hospital stroke (IHS) including the reason for hospitalization and invasive procedures before the stroke. We aimed to extend current knowledge.

**Patients and methods:**

All adult patients with IHS in Sweden during 2010–2019 registered in the Swedish Stroke Register (Riksstroke) were included. The cohort was cross-linked to the National Patient Register and data extracted on background diagnoses, main discharge diagnoses, and procedure codes for the hospitalization when IHS occurred and any hospital-based healthcare contacts within 30 days before IHS.

**Results:**

231,402 stroke cases were identified of which 12,551 (5.4%) were in-hospital and had corresponding entries in the National Patient Register. Of the IHS patients, 11,420 (91.0%) had ischemic stroke and 1131 (9.0%) hemorrhagic stroke; 5860 (46.7%) of the IHS patients had at least one invasive procedure prior to ictus. 1696 (13.5%) had a cardiovascular procedure and 560 (4.5%) a neurosurgical procedure. 1319 (10.5%) patients only had minimally invasive procedures such as blood product transfusion, hemodialysis, or central line insertion. Common discharge diagnosis in patients with no invasive procedures were cardiovascular disorders, injuries, and respiratory disorders.

**Discussion and conclusion:**

One in every 17 strokes in Sweden occur in a hospital. In this unselected large cohort the previously reported major causes for in-hospital stroke, cardiovascular and neurosurgical procedures, preceded IHS in only 18.0% of cases suggesting that other etiologies are more common than previously reported. Future studies should aim at determining absolute risks of stroke after surgical procedures and ways of risk reduction.

## Introduction

In-hospital stroke (IHS) is defined as a cerebrovascular insult that occurs during hospitalization in an already admitted patient. IHS accounts for 2.2%–16.6% of all strokes.^1–9^ Few studies have reported the primary reason for hospitalization and invasive procedures preceding the stroke among these patients.^2,5,9–11^ Among the *n* = 1499^2,5,9–11^ previously reported patients 46%–69% had undergone an invasive procedure prior to their stroke, most commonly cardiac or neurosurgical procedures.^2,5,10,11^

The aim of this study was to present the characteristics of patients with IHS including the main reason for hospitalization and invasive procedures performed before the stroke in a nation-wide, decade-long Swedish cohort of patients with IHS divided into ischemic and hemorrhagic stroke.

## Methods

### Design and setting

The Swedish stroke register (Riksstroke) was used to define the cohort. All registered patients >18 years old with International Classification of Diseases (ICD-10) diagnoses I61 (hemorrhagic stroke), I63 (ischemic stroke), and I64 (unspecified stroke) that were already hospitalized when the stroke occurred were included. Patients were included from 2010 (when the IHS variable was introduced) until 2019. Data extraction was performed in February 2021. Data regarding the hospitalization during which the stroke occurred and any hospitalizations or visits to hospital-based outpatient clinics within 30 days prior to the in-hospital stroke were retrieved by individual cross-linkage with the National Patient Register.^12^

Sweden is inhabited by roughly 10 million people. The healthcare system is tax funded and managed by 21 separate counties. In 2021, there were ~1.4 million hospital discharges, 13 million visits to hospital outpatient clinics, and 24,000 stroke cases in Sweden.^13^

### Data sources

Riksstroke is a quality register in which all hospitals in Sweden admitting acute stroke patients participate (*n* = 72 in 2021). Data is collected prospectively on the quality of acute care and secondary prevention as well as on rehabilitation and outcomes. The coverage has been estimated to be 90%.^14^

The National Patient Register consists of the inpatient and outpatient registers managed by the National Board of Health and Welfare.^12^ It has a >99% coverage of all somatic hospital discharges but the outpatient register coverage is lower than that due to missing data from private caregivers.^15^ Visits to primary care are not registered.

### Variables

#### Patient characteristics

Riksstroke provided information on comorbidities (hypertension, previous stroke, previous transitory ischemic attack/amaurosis fugax, atrial fibrillation and diabetes), smoking status, prescribed medications (lipid-lowering drugs, antihypertensives, antiplatelets, and anticoagulants), hospital type at stroke onset, functional status before stroke (activities in daily living (ADL)) and stroke severity (level of consciousness at presentation). The reporting hospitals were nine university hospitals, 22 specialized non-university hospitals, and 41 community hospitals.^16^

Additional comorbidities (congestive heart failure, chronic obstructive pulmonary disease, myocardial infarction, renal failure, neoplasia, prosthetic heart valve, and other cardiac devices) were extracted from the National Patient Register. A disease was considered present if any of the corresponding ICD-10 codes appeared in the National Patient Register at least once within 5 years before the stroke.

#### Invasive procedures

All procedure codes were extracted from each contact with health care services within 30 days prior to the stroke, including procedures before the stroke but during the in-hospital stay. Procedure codes were not linked to a specific hospital; therefore, procedures could have been performed at a different hospital than where the stroke occurred.

Procedure codes in the National Patient Register are classified according to the Swedish Classification of Care Measures, which is subdivided into surgical and nonsurgical codes. Most surgical procedure codes are invasive while only a minority of the non-surgical procedure codes are. All procedure codes occurring within 30 days prior to stroke onset were manually classified as invasive/noninvasive during author group discussions (codes available on demand). Procedure codes related to treatment of acute ischemic stroke (e.g. endovascular thrombectomy) were excluded. Procedures occurring on the same day as the stroke were excluded in a sensitivity analysis due to possible reverse causation.

Procedures were categorized as cardiovascular, neurosurgical, pulmonary, gastrointestinal, urological, orthopedic, endoscopic, or “other surgeries,” the latter including ear-nose-throat, ophthalmological, endocrine, gynecological, obstetric, and dental procedures that were too rare to classify. Each case could have had surgical procedures from multiple of the aforementioned categories. A final category, “minimally invasive procedures,” was assigned to all other invasive procedure codes not fitting into any of the other categories and not representing surgery per se, such as establishment of a central arterial line access or transfusion of blood products. Patients in this category had not undergone any procedures from the previous categories.

#### Main discharge diagnoses

For every hospital discharge the cause of admission should be registered as the main discharge diagnosis in the National Patient Register; unless when a condition that is more resource-consuming develops during the hospital stay in which case the latter is registered as the main discharge diagnosis.

The main discharge diagnoses were extracted from each contact with healthcare within 30 days prior to the stroke and classified into categories according to the ICD-10 chapters. The main discharge diagnosis registered on the hospitalization where the in-hospital stroke occurred was interpreted as the reason for being admitted (stroke diagnoses excluded).

### Statistical analysis

Data was primarily analyzed by descriptive statistics. To assess relevant patient group imbalances, we used standardized differences (*d*) of means (continuous variables) or prevalence (binary variables).^17^ Absolute values of *d* > 0.1 were considered as relevant differences. Students *t*-test (for continuous) and Pearson χ^2^-test (for categorical variables) were used for formal group comparisons. A difference with a *p*-value <0.05 was considered statistically significant; *p*-values were not adjusted for multiple testing. We used IBM SPSS Statistics for Mac, Version 28 (Armonk, NY) for statistical analyses.

## Results

**Figure 1.**
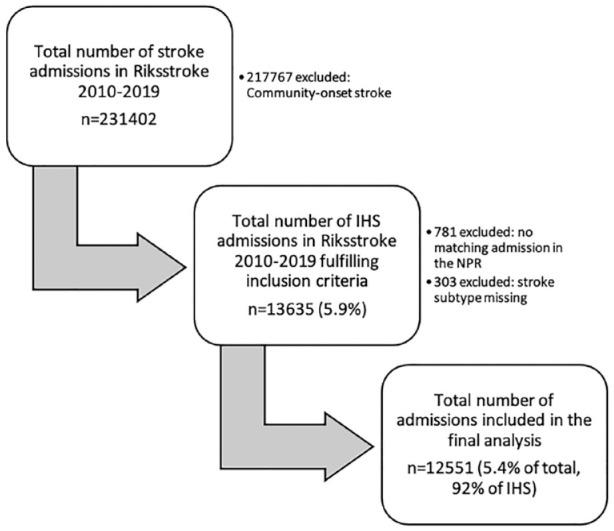
Flow chart of patient inclusion/exclusion. IHS: in-hospital stroke; NPR: National Patient Register.

Of the 231,402 patients that were registered in Riksstroke during the study period, 13,635 (5.9%) were IHS. Of these, 781 were excluded due to no corresponding admission found in the National Patient Register, and 303 were excluded due to stroke subtype missing, leaving 12,551 IHS patients (Figure 1).

### IHS patient characteristics

**Figure 2.**
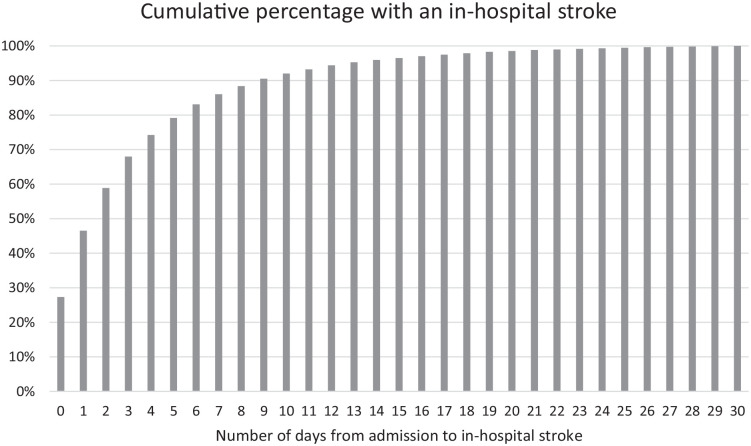
Bar chart showing the daily cumulative percentage of patients with an IHS after hospital admission.

The mean age of IHS patients was 76.3 years; 50.8% were female (6377/12,551), and 65.5% had hypertension (8126/12,401), which was the most common comorbidity (Table 1). A majority of IHS patients (91.0%; 11,420/12,551) had ischemic strokes and had no healthcare-contacts within 30 days prior to the hospitalization during which the stroke occurred (62.9%; 7891/12,551). Patients with ischemic stroke were older and had a higher proportion with atrial fibrillation compared to hemorrhagic stroke. IHS occurred in median on day 1 (IQR day 0–4) after admission (Figure 2).

**Table 1.** Patient characteristics prior to admission in *n* = 12,551 in-hospital stroke patients divided by stroke type.

	Stroke type		Standardized difference (*d*)	*p*	Total (*n* = 12 551)	Missing
	Ischemic (*n* = 11,420)	Hemorrhagic (*n* = 1131)				
Age, mean (SD) years	76.8 (11.9)	72.1 (14.3)	−0.39	<0.001	76.3 (12.2)	0 (0.0%)
Female sex, *n* (%)	5823 (51.0%)	554 (49.0%)	0.03	0.198	6377 (50.8%)	0 (0.0%)
ADL independency, *n* (%)	9256 (86.0%)	871 (83.6%)	0.06	0.002	10,127 (85.8%)	748 (6.0%)
Level of consciousness at presentation				<0.001		745 (5.9%)
Awake and alert	8318 (77.4%)	651 (61.5%)	0.29		8969 (76.0%)	
Drowsy or unconscious	2430 (22.6%)	407 (38.5%)	−0.29		2837 (24.0%)	
Hospital-type				<0.001		0 (0.0%)
*University hospital*	2466 (21.6%)	300 (26.5%)	−0.09		2788 (22.0%)	
*Specialized non-university hospital*	5156 (45.1%)	460 (40.7%)	0.07		5616 (44.7%)	
*Community hospital*	3798 (33.3%)	371 (32.8%)	0.01		4169 (33.2%)	
Hypertension, *n* (%)	7468 (66.1%)	658 (59.5%)	0.11	<0.001	8126 (65.5%)	150 (1.2%)
Diabetes mellitus, *n* (%)	2854 (25.1%)	244 (21.8%)	0.06	0.015	3098 (24.8%)	84 (0.7%)
Smoking, *n* (%)	1244 (12.6%)	107 (11.6%)	0.02	0.354	1351 (12.5%)	1782 (14.2%)
Atrial fibrillation, *n* (%)	4560 (40.3%)	299 (26.9%)	0.24	<0.001	4859 (39.1%)	138 (1.1%)
Congestive heart failure, *n* (%)	1514 (13.3%)	123 (10.9%)	0.06	0.023	1637 (13.0%)	0 (0.0%)
Prosthetic heart valve, *n* (%)	103 (0.9%)	22 (1.9%)	−0.07	<0.001	125 (1.0%)	0 (0.0%)
Other intracardiac device, *n* (%)	167 (1.5%)	17 (1.5%)	0.00	0.880	184 (1.5%)	0 (0.0%)
Previous stroke, *n* (%)	2234 (19.7%)	242 (21.7%)	−0.04	0.120	2476 (19.9%)	117 (0.9%)
Previous TIA/amaurosis, *n* (%)	986 (8.8%)	69 (6.5%)	0.07	0.004	1055 (8.5%)	184 (1.5%)
Previous myocardial infarction, *n* (%)	1478 (12.9%)	119 (10.5%)	0.06	0.020	1597 (12.7%)	0 (0.0%)
Peripheral vascular disease, *n* (%)	819 (7.2%)	62 (5.5%)	0.06	0.034	881 (7.0%)	0 (0.0%)
Cancer last 5 years, *n* (%)	2269 (19.9%)	278 (24.6%)	−0.09	<0.001	2547 (20.3%)	0 (0.0%)
Metastases, *n* (%)	235 (2.1%)	44 (3.9%)	−0.09	<0.001	279 (2.2%)	0 (0.0%)
Pulmonary embolism or deep vein thrombosis, *n* (%)	434 (3.8%)	77 (6.8%)	−0.11	<0.001	511 (4.1%)	0 (0.0%)
Chronic obstructive pulmonary disease, *n* (%)	503 (4.4%)	45 (4.0%)	0.02	0.504	548 (4.4%)	0 (0.0%)
Renal failure, *n* (%)	566 (5.0%)	83 (7.3%)	−0.08	<0.001	649 (5.2%)	0 (0.0%)
Treated with any anticoagulant agent, *n* (%)	1703 (15.1%)	219 (19.9%)	−0.11	<0.001	1922 (15.5%)	153 (1.2%)
Treated with any antithrombotic agent, *n* (%)	4381 (38.8%)	311 (28.2%)	0.18	<0.001	4692 (37.9%)	159 (1.3%)
Treated with statins, *n* (%)	3630 (31.8%)	334 (29.5%)	0.04	<0.001	3964 (31.6%)	180 (1.4%)
Hospitalization within 30 days prior to stroke, *n* (%)	2972 (26.0%)	334 (29.5%)	−0.06	0.011	3306 (26.3%)	0 (0.0%)
Visit to hospital outpatient clinic within 30 days before stroke, *n* (%)	1803 (15.8%)	115 (10.2%)	0.13	<0.001	1918 (15.3%)	0 (0.0%)
Invasive procedure within 30 days, *n* (%)	5368 (47.0%)	492 (43.5%)	0.06	0.025	5860 (46.7%)	0 (0.0%)

ADL= activities in daily living; SD=standard deviation.

### Invasive procedures and discharge diagnoses

46.7% (5860/12,551) of the in-hospital stroke patients had at least one invasive procedure performed within 30 days before the stroke. The time from any invasive procedure to IHS was in median 1 day (IQR 0–4 days). Having an invasive procedure prior to stroke was more common in university hospitals compared to non-university hospitals (53.7% vs 44.7%). The most common categories of procedures were cardiovascular, orthopedic, endoscopic, and neurosurgical (Table 2). The most common types of procedures in each category are presented in Supplemental Table 1. For patients with a non-stroke discharge diagnosis who had no procedures performed, the most common diagnoses were non-stroke circulatory system disorders, injuries, and respiratory disorders (Table 2).

**Table 2.** Estimation of stroke etiology presented as the proportion of procedures performed among patients undergoing procedures and the main discharge diagnosis for all patients. The sum of procedure types is exceeding the number who had invasive procedures due to some patients having more than one procedure.

	*N* = 12,551 in-hospital stroke patients of which *n* = 5860 had an invasive procedure	
Procedure types		
Cardiovascular procedure	1696 (13.5%)	
Orthopedic procedure	1015 (8.1%)	
Endoscopy	821 (6.5%)	
Gastrointestinal procedure	576 (4.6%)	
Neurosurgical procedure	560 (4.5%)	
Pulmonary procedure	319 (2.5%)	
Other surgeries	238 (1.9%)	
Urological procedure	172 (1.4%)	
Minimally invasive procedures	1319 (10.5%)	
ICD-10 diagnosis group	Invasive procedures *n* = 5860	No invasive procedures *n* = 6691
Stroke	1582 (27.0%)	3655 (54.6%)
Non-stroke circulatory system	1695 (39.6%)	1025 (33.8%)
Injuries	810 (18.9%)	349 (11.5%)
Neoplasia	455 (10.6%)	215 (7.1%)
Respiratory	184 (4.3%)	300 (10.0%)
Digestive system	333 (7.8%)	138 (4.5%)
Musculoskeletal	220 (5.1%)	106 (3.5%)
Symptoms	88 (2.1%)	190 (6.3%)
Genitourinary system	119 (2.8%)	135 (4.4%)
Infectious	104 (2.4%)	147 (4.8%)
Neurological	74 (1.7%)	109 (3.6%)
Other	181 (4.2%)	257 (8.5%)

### Sensitivity analysis

When excluding procedures performed the same day the stroke occurred, the proportion of patients who had undergone at least one invasive procedure within 30 days prior to the stroke decreased from 46.7% (5860/12,551) to 35% (4394/12,551). No relevant differences were detected in the distribution of procedure types (Supplemental Figure 1).

## Discussion

In this study, we present the characteristics of a decade-long, nation-wide cohort of over 12,000 IHS cases in Sweden including main discharge diagnoses and invasive procedures performed within 30 days prior to the stroke.

One of our main findings was that almost half of the IHS patients had undergone at least one invasive procedure within 30 days before their stroke. This is low compared with previous reports, despite the fact that this study unlike earlier ones included minimally invasive procedures and investigated a period of 30 days before the IHS. The nationwide catchment area and inclusion of non-university hospitals may partially account for these differences. The previously apparent major risk factors for IHS (cardiovascular and neurosurgical procedures) preceded the IHS in a lower proportion compared to earlier publications where such data was available,^2,5,11^ suggesting that other procedures preceding IHS are more important than previously believed. A majority of the IHS patients in our material had not had any invasive procedures performed before the stroke, suggesting that other factors in admitted patients also influence the risk for stroke.

Just over one fifth of the patients who had undergone at least one invasive procedure prior to stroke did not have surgery, but merely had transfusion of blood products, hemodialysis, intubation and/or respiratory support, central or arterial line insertion, or intravenous administration of pharmaceutical agents. Whether this finding suggests that such procedures do increase the risk for stroke – or merely that critically ill patients in need of these interventions are at an increased risk for stroke – cannot be deduced from the data. Previous studies have not included such minimally invasive procedures and this group is presented separately to allow for comparison with other studies. To investigate the risk for post procedural stroke per group of procedures, future studies should if possible aim to include the total populations undergoing each group of procedures.

We found that over half of the IHS patients without invasive procedures had ischemic or hemorrhagic stroke registered as their main discharge diagnosis, and we were unfortunately not able to further estimate what caused their hospitalization. These diagnoses were not assumed to be the main reasons for admission. Among patients without invasive procedures nor a stroke diagnosis as main discharge diagnosis, non-stroke circulatory system disorders were most common followed by injuries and respiratory disorders.

Dividing the cohort based on hospital type revealed only slight differences in background factors (Supplemental Table 3). This cohort had a majority of cases from non-university hospitals, which should give a fair picture of the in-hospital stroke population on a nationwide level. When excluding non-university hospitals the proportion of patients with an invasive procedure preceding the IHS in our data rises to levels in the vicinity of those in earlier reports (Supplemental Table 4).

### Strengths and limitations

The major strengths of this study are the high coverage of stroke cases from Riksstroke^14^ and the high coverage of hospital-based health care contacts in the National Patient Register including all hospitals from comprehensive stroke-care units to community hospitals. Furthermore, this is a long-term cohort and by far the largest cohort of IHS patients with data that estimate the reasons for hospitalization prior to IHS.

One limitation is that the National Patient Register only records diagnoses from hospitals. Background diagnoses only registered in primary care and contacts with primary care within 30 days of stroke are not covered. The impact of this is somewhat mitigated due to the rarity of invasive procedures in primary care. Furthermore, the study was limited by the fact that we did not have admission diagnoses per se, but rather relied on the main discharge diagnosis to decide what was the main reason for admission. Even if this study showed that a significant group of IHS patients had an invasive procedure in a close temporal relation to stroke we cannot be certain that the procedure caused the stroke. We also cannot exclude that residual confounding, such as the possibility of an increased cardiovascular risk factor load among patients in need of surgery, may bias the results. Lastly, the aspect of register studies being limited to the quality of data being inserted into the registers also deserves to be mentioned. However, the National Patient Register undergoes regular validations and takes measures to reduce missing data and increase data quality.^12^ Regarding Riksstroke, data quality is good and is maintained through regular validations and continuous quality improvement.^14^ We believe that our results could be generalized to countries with demographics and healthcare organizations similar to Sweden’s.

To conclude, one out of every 17 strokes in Sweden occurs in a hospital. Common groups of invasive procedures preceding in-hospital strokes include cardiovascular, neurosurgical, and orthopedic procedures but a large proportion of in-hospital strokes are not preceded by any procedures. Common causes of admission in patients with no invasive procedures are non-stroke cardiovascular disorders, injuries, and respiratory disorders.

## Supplemental Material

sj-docx-1-eso-10.1177_23969873231182761 – Supplemental material for Characteristics of in-hospital stroke patients in Sweden: A nationwide register-based study Click here for additional data file. Supplemental material, sj-docx-1-eso-10.1177_23969873231182761 for Characteristics of in-hospital stroke patients in Sweden: A nationwide register-based study by Ilan Ben-Shabat, David Darehed, Marie Eriksson and Jonatan Salzer in European Stroke Journal
