# Supplementary material for: Characteristics of in-hospital stroke patients in Sweden: A nationwide register-based study
Source: Eur Stroke J. 2023 Jun 17;8(3):777–83. doi: 10.1177/23969873231182761 (PMC10472946; doi:10.1177/23969873231182761)
Supplement: sj-docx-1-eso-10.1177_23969873231182761 – Supplemental material for Characteristics of in-hospital stroke patients in Sweden: A nationwide register-based study [file sj-docx-1-eso-10.1177_23969873231182761.docx]

***Supplementary figure 1:*** Comparing categories of invasive procedures performed within 30 days with and without the same day as stroke included. Each case could have had surgical procedures from more than one of the categories.

***Supplementary table 1:*** Most frequent procedure codes occurring in each procedure group within 30 days prior to in-hospital stroke.

| *Procedure group* |
| --- |
| **Cardiovascular** |
| - *Coronary angiography with/without angioplasty* |
| - *Total cardiopulmonary bypass* |
| - *Coronary artery bypass surgery* |
| - *Valvular surgery* |
| - *Aortic root surgery* |
| - *Pacemaker insertion or use of temporary pacemaker* |
| **Orthopedic** |
| - *Hip and thigh surgery* |
| - *Knee and leg surgery* |
| **Endoscopy** |
| - *Gastroscopy* |
| - *Colonoscopy or enteroscopy* |
| - *Bronchoscopy* |
| **Gastrointestinal** |
| - *Lower gastrointestinal surgery* |
| - *Upper gastrointestinal surgery* |
| **Neurosurgical** |
| - *Open cranial or intracranial surgery* |
| - *Diagnostic procedures* |
| - *Endovascular intracranial procedures (endovascular thrombectomy for ischemic stroke excluded)* |
| **Pulmonary** |
| - *Pleural, diaphragm, or chest wall surgery* |
| - *Bronchotracheal surgery* |
| **Other surgeries** |
| - *Skin procedures* |
| - *Ear-nose-throat-procedures* |
| - *Gynecological surgery* |
| **Urological** |
| - *Ureter or bladder surgery* |
| - *Kidney surgery* |
| **Minimally invasive procedures** |
| - *Administration of pharmaceutical agent (mostly intravenous)* |
| - *Transfusion of blood products* |
| - *Hemodialysis* |
| - *Respirator/intubation/ventilation* |
| - *Central venous access or arterial line* |

***Supplementary table 2:*** *Most common main discharge diagnoses (ICD-10) for the whole cohort of IHS cases.*

| **Diagnosis** | **ICD-10 code** | **Total (n=12551)** |
| --- | --- | --- |
| Acute myocardial infarction | I21 | 583 |
| Fracture of femur | S72 | 548 |
| Aortic aneurysm and dissection | I71 | 278 |
| Nonrheumatic aortic valve disorders | I35 | 248 |
| Heart failure | I50 | 240 |
| Angina pectoris | I20 | 189 |
| Atrial fibrillation and flutter | I48 | 187 |
| Bacterial pneumonia, not elsewhere classified | J15 | 124 |
| Pneumonia, organism unspecified | J18 | 124 |
| Intracranial injury | S06 | 105 |
| Other sepsis | A41 | 104 |
| Occlusion and stenosis of precerebral arteries not resulting in cerebral infarction | I65 | 104 |
| Cerebral aneurysm, nonruptured | I67 | 90 |
| Arterial embolism and thrombosis | I74 | 88 |
| Atherosclerosis | I70 | 85 |
| Acute and subacute endocarditis | I33 | 80 |
| Coxarthrosis | M16 | 78 |
| Transient cerebral ischemic attacks and related syndromes | G45 | 74 |
| Chronic ischemic heart disease | I25 | 74 |
| Other disorders of urinary system | N39 | 70 |
| Malignant neoplasm of colon | C18 | 68 |
| Complications of procedures, not elsewhere classified | T81 | 66 |
| Malignant neoplasm of bronchus and lung | C34 | 65 |
| Other diseases of digestive system | K92 | 62 |
| Complications of internal orthopedic prosthetic devices, implants and grafts | T84 | 55 |
| Pulmonary embolism | I26 | 52 |
| Paralytic ileus and intestinal obstruction without hernia | K56 | 50 |
| Other chronic obstructive pulmonary disease | J44 | 46 |
| Fracture of lumbar spine and pelvis | S32 | 43 |
| Other and unspecified nontraumatic intracranial hemorrhage | I62 | 42 |
| Other spondylopathies | M48 | 42 |
| Cholelithiasis | K80 | 41 |
| Acute tubulo-interstitial nephritis | N10 | 40 |
| Crushing injury and traumatic amputation of part of abdomen, lower back and pelvis | S82 | 40 |
| Gonarthrosis | M17 | 39 |
| Acute renal failure | N17 | 39 |
| Gastric ulcer | K25 | 35 |
| Duodenal ulcer | K26 | 35 |
| Cardiomyopathy | I42 | 34 |
| Complications of cardiac and vascular prosthetic devices, implants and grafts | T82 | 34 |
| Nonrheumatic mitral valve disorders | I34 | 33 |
| Fracture of shoulder and upper arm | S42 | 33 |
| Abdominal and pelvic pain | R10 | 32 |
| Chronic kidney disease | N18 | 31 |
| Other surgical follow-up care | Z48 | 31 |
| Diverticular disease of intestine | K57 | 30 |
| Superficial injury of hip and thigh | S70 | 30 |
| Malignant neoplasm of bladder | C67 | 29 |
| Respiratory failure, not elsewhere classified | J96 | 29 |
| Fracture of rib(s), sternum and thoracic spine | S22 | 29 |
| Type 2 diabetes mellitus | E11 | 27 |
| Malignant neoplasm of pancreas | C25 | 26 |
| Other bacterial intestinal infections | A04 | 25 |
| Malignant neoplasm of brain | C71 | 25 |
| Acute pancreatitis | K85 | 25 |
| Neoplasm of uncertain or unknown behavior of oral cavity and digestive organs | D37 | 24 |
| Neoplasm of uncertain or unknown behavior of brain and central nervous system | D43 | 24 |
| Cardiac arrest | I46 | 24 |
| Benign neoplasm of meninges | D32 | 23 |
| Depressive episode | F32 | 23 |
| Pain in throat and chest | R07 | 23 |
| Secondary malignant neoplasm of respiratory and digestive organs | C78 | 22 |
| Other cardiac arrhythmias | I49 | 22 |
| Pneumonitis due to solids and liquids | J69 | 22 |
| Unspecified hematuria | R31 | 22 |
| Malignant neoplasm of rectum | C20 | 21 |
| Malignant neoplasm of prostate | C61 | 21 |
| Sequelae of cerebrovascular disease | I69 | 21 |
| Pleural effusion, not elsewhere classified | J90 | 21 |
| Fracture of neck | S12 | 21 |
| Medical observation and evaluation for suspected diseases and conditions, ruled out | Z03 | 21 |
| Atrioventricular and left bundle-branch block | I44 | 20 |
| Other aneurysm and dissection | I72 | 20 |
| Other medical care | Z51 | 20 |

***Supplementary table 3:*** Background factors for the whole cohort of IHS cases divided by hospital type (university hospital vs. non-university hospital).

|  | **Hospital type** | |  |  |  |  |
| --- | --- | --- | --- | --- | --- | --- |
|  | ***University hospital n=2766*** | ***Non-university hospital n=9785*** | ***Standardized difference (d)*** | ***p*** | ***Total (n=12551)*** | ***Missing*** |
| Age, mean (SD) years | 74 (14) | 77 (12) | -0.29 | <0.001 | 76 (12) | 0 (0.0%) |
| Female sex n (%) | 1352 (48.9%) | 5025 (51.4%) | -0.04 | 0.022 | 6377 (50.8%) | 0 (0.0%) |
| ADL independency, n (%) | 2348 (89.3%) | 7779 (84.8%) | 0.11 | <0.001 | 10127 (85.8%) | 745 (5.9%) |
| Stroke type |  |  |  | <0.001 |  |  |
| - Ischemic | 2466 (89.2%) | 8954 (91.5%) | -0.06 |  | 11420 (91.0%) |  |
| - Hemorrhagic | 300 (10.8%) | 831 (8.5%) | 0.06 |  | 1131 (9.0%) |  |
| Level of consciousness at presentation |  |  |  | <0.001 |  | 822 (6.5%) |
| - Awake and alert | 1890 (70.8%) | 7079 (77.5%) | -0.12 |  | 8969 (76.0%) |  |
| - Drowsy or unconscious | 778 (29.2%) | 2059 (22.5%) | 0.12 |  | 2837 (24.0%) |  |
| Hypertension, n (%) | 1652 (61.0%) | 6474 (66.8%) | -0.1 | <0.001 | 8126 (65.5%) | 150 (1.8%) |
| Diabetes mellitus, n (%) | 647 (23.8%) | 2451 (25.2%) | -0.03 | 0.139 | 3098 (24.8%) | 84 (2.6%) |
| Smoking, n (%) | 330 (14.3%) | 1021 (12.1%) | 0.05 | 0.005 | 1351 (12.5%) | 1782 (56.9%) |
| Atrial fibrillation, n (%) | 995 (36.7%) | 3864 (39.8%) | -0.05 | 0.003 | 4859 (39.1%) | 138 (2.8%) |
| Congestive heart failure, n (%) | 361 (13.1%) | 1276 (13.0%) | 0.00 | 1.000 | 1637 (13.0%) | 0 (0.0%) |
| Prosthetic heart valve, n (%) | 42 (1.5%) | 83 (0.8%) | 0.05 | 0.003 | 125 (1.0%) | 0 (0.0%) |
| Other intracardiac device, n (%) | 44 (1.6%) | 140 (1.4%) | 0.01 | 0.533 | 184 (1.5%) | 0 (0.0%) |
| Previous stroke, n (%) | 523 (19.3%) | 1953 (20.1%) | -0.02 | 0.342 | 2476 (19.9%) | 117 (4.5%) |
| Previous TIA/amaurosis, n (%) | 226 (8.4%) | 829 (8.6%) | -0.01 | 0.753 | 1055 (8.5%) | 184 (14.9%) |
| Previous myocardial infarction, n (%) | 362 (13.1%) | 1235 (12.6%) | 0.01 | 0.519 | 1597 (12.7%) | 0 (0.0%) |
| Peripheral vascular disease, n (%) | 214 (7.7%) | 667 (6.8%) | 0.03 | 0.099 | 881 (7.0%) | 0 (0.0%) |
| Cancer last 5 years, n (%) | 614 (22.2%) | 1933 (19.8%) | 0.05 | 0.005 | 2547 (20.3%) | 0 (0.0%) |
| Metastases, n (%) | 90 (3.3%) | 189 (1.9%) | 0.07 | <0.001 | 279 (2.2%) | 0 (0.0%) |
| Pulmonary embolism or deep vein thrombosis, n (%) | 130 (4.7%) | 381 (3.9%) | 0.03 | 0.062 | 511 (4.1%) | 0 (0.0%) |
| Chronic obstructive pulmonary disease, n (%) | 128 (4.6%) | 420 (4.3%) | 0.01 | 0.462 | 548 (4.4%) | 0 (0.0%) |
| Renal failure, n (%) | 158 (5.7%) | 491 (5.0%) | 0.03 | 0.144 | 649 (5.2%) | 0 (0.0%) |
| Treated with any anticoagulant agent, n (%) | 420 (15.5%) | 1502 (15.5%) | 0.00 | 0.954 | 1922 (15.5%) | 153 (7.4%) |
| Treated with any antithrombotic agent, n (%) | 943 (34.8%) | 3749 (38.7%) | -0.07 | <0.001 | 4692 (37.9%) | 159 (3.3%) |
| Treated with statins, n (%) | 820 (29.6%) | 3144 (32.1%) | -0.04 | <0.001 | 3964 (31.6%) | 180 (1.4%) |
| Hospitalization within 30 days prior to stroke, n (%) | 776 (28.1%) | 2530 (25.9%) | 0.04 | 0.021 | 3306 (26.3%) | 0 (0.0%) |
| Visit to hospital outpatient clinic within 30 days before stroke, n (%) | 447 (16.2%) | 1471 (15.0%) | 0.03 | 0.150 | 1918 (15.3%) | 0 (0.0%) |
| Invasive procedure within 30 days, n (%) | 1486 (53.7%) | 4374 (44.7%) | 0.15 | <0.001 | 5860 (46.7%) | 0 |

**Supplementary table 4**: Invasive procedures performed within 30 days of in-hospital stroke divided by hospital type (university hospital vs non-university hospital). Each case could have had surgical procedures from more than one of the categories.

|  | University hospital n=2766 | Non-university hospital n=9785 |
| --- | --- | --- |
| Invasive procedure | 1486 (53.7%) | 4374 (44.7%) |
| Cardiovascular | 476 (17.2%) | 1220 (12.5%) |
| Orthopedic | 177 (6.4%) | 838 (8.6%) |
| Endoscopy | 230 (8.3%) | 591 (6.0%) |
| Gastrointestinal | 152 (5.5%) | 424 (4.3%) |
| Neurosurgical | 203 (7.3%) | 357 (3.6%) |
| Pulmonary | 86 (3.1%) | 233 (2.4%) |
| Other surgeries | 75 (2.7%) | 163 (1.7%) |
| Urological | 49 (1.8%) | 123 (1.3%) |
| Minimally invasive procedures | 314 (11.4%) | 1005 (10.3%) |
